# A Knowledge-Driven Approach to Extract Disease-Related Biomarkers from the Literature

**DOI:** 10.1155/2014/253128

**Published:** 2014-04-16

**Authors:** À. Bravo, M. Cases, N. Queralt-Rosinach, F. Sanz, L. I. Furlong

**Affiliations:** Research Programme on Biomedical Informatics (GRIB), Hospital del Mar Medical Research Institute (IMIM), Department of Experimental and Health Sciences, Universitat Pompeu Fabra, C/Dr Aiguader 88, E-08003 Barcelona, Spain

## Abstract

The biomedical literature represents a rich source of biomarker information. However, both the size of literature databases and their lack of standardization hamper the automatic exploitation of the information contained in these resources. Text mining approaches have proven to be useful for the exploitation of information contained in the scientific publications. Here, we show that a knowledge-driven text mining approach can exploit a large literature database to extract a dataset of biomarkers related to diseases covering all therapeutic areas. Our methodology takes advantage of the annotation of MEDLINE publications pertaining to biomarkers with MeSH terms, narrowing the search to specific publications and, therefore, minimizing the false positive ratio. It is based on a dictionary-based named entity recognition system and a relation extraction module. The application of this methodology resulted in the identification of 131,012 disease-biomarker associations between 2,803 genes and 2,751 diseases, and represents a valuable knowledge base for those interested in disease-related biomarkers. Additionally, we present a bibliometric analysis of the journals reporting biomarker related information during the last 40 years.

## 1. Introduction

The Biomarkers Definition Working Group (formed by the US National Institutes of Health (NIH) and the US Food and Drug Administration (FDA), academia, and industry) defined biomarker as “*a characteristic that is objectively measured and evaluated as an indicator of normal biologic processes, pathogenic processes, or pharmacologic responses to a therapeutic intervention*” [1]. With the advent of the genomics era, in April 2008, the FDA published in one of its “Guidance for Industry” documentations the specific definition of a genomic biomarker as “*a measurable DNA and/or RNA characteristic that is an indicator of normal biologic processes, pathogenic processes, and/or response to therapeutic or other interventions*” [2]. More recently, Anderson and Kodukula [3] provided some definitions of different types of biomarkers (e.g., surrogate, clinical endpoint, diagnostic, prognostic, predictive, pharmacodynamic, efficacy, and toxicity/safety [4–6]) within their review of the role of biomarkers in pharmacology and drug discovery. All these definitions specify the requirements to be held by a biomarker, the different types that exist, their potential role in disease diagnosis and progression or in the therapeutic response control, and their utility for the assessment of new chemical entities as potential lead therapeutics [3].

Thousands of biomolecules are being investigated as potential biomarkers, but most of them do not advance effectively for diagnostic, prognostic, or therapeutic goals for different reasons (for a detailed discussion on this topic see [7–9]). The results of the research on potential biomarkers are widely reported on the biomedical literature. The MEDLINE database [10] has currently indexed more than 23 M articles, and since 1989 the MeSH term “Biological Markers” is applied to annotate those articles that provide data on “*measurable and quantifiable biological parameters (e.g., specific enzyme concentration, specific hormone concentration, specific gene phenotype distribution in a population, presence of biological substances) which serve as indices for health- and physiology-related assessments, such as disease risk, psychiatric disorders, environmental exposure and its effects, disease diagnosis, metabolic processes, substance abuse, pregnancy, cell line development, epidemiologic studies, etc*.” [11], and later on, in 2008, the MeSH term “Biomarkers, Pharmacological” was introduced to specifically annotate the “*measurable biological parameters that serve for drug development, safety and dosing (DRUG MONITORING)*” [12]. In particular, genomic biomarkers are frequently reported in the literature together with disease-related information. Thus, the MEDLINE database contains valuable knowledge for those interested in gathering information on biomarkers. In order to identify, extract, and analyse this information from the literature, automatic processing of the texts is required [13]. There are only few reports on text mining approaches in the biomarkers field [14–16]. Here, we present a knowledge-driven text mining approach for the extraction of disease-related biomarker information from the literature. Our approach, firstly, takes advantage of biomarker-specific MeSH terms annotations to retrieve a specific and comprehensive pool of publications from MEDLINE, secondly, applies our named entity recognition method (BioNER) to  (1) identify genes and diseases as entities of interest, (2) filter ambiguous entities, (3) cluster equivalent terms to a certain concept, (4) characterize those genes as potential biomarkers based on terminology used, and, finally, (5) find associations between genes and diseases in single sentences, and ranks the associations based on their frequency in the literature. This approach, that allows the unique identification of genomic biomarkers and their associated diseases, was applied to the MEDLINE database resulting in a comprehensive knowledge base on disease-related biomarkers, which is publicly available at http://ibi.imim.es/biomarkers/. In addition, we provide an analysis of the results obtained and present an evaluation of the trend of biomarker research reporting as a topic in the scientific literature.

## 2. Material and Methods 

We developed a text mining workflow aimed at extracting information on disease-related biomarkers from scientific publications. Briefly, after document selection, the text mining approach comprises as a first step the recognition and normalization of the* disease* and* biomarker* entities in biomedical publications by means of the biomedical named entity recognition (BioNER) system and, secondly, the identification of relationships between the aforementioned entities by their cooccurrence in sentences. For example, the following sentence (taken from PMID: 17397492), “**CK20** *is an important biomarker that can be used to identify* **TCC** *in urine cytology smears,*” contains the cooccurrence of the entities ** CK20** (gene) and ** TCC** (disease).

**Figure 1:**
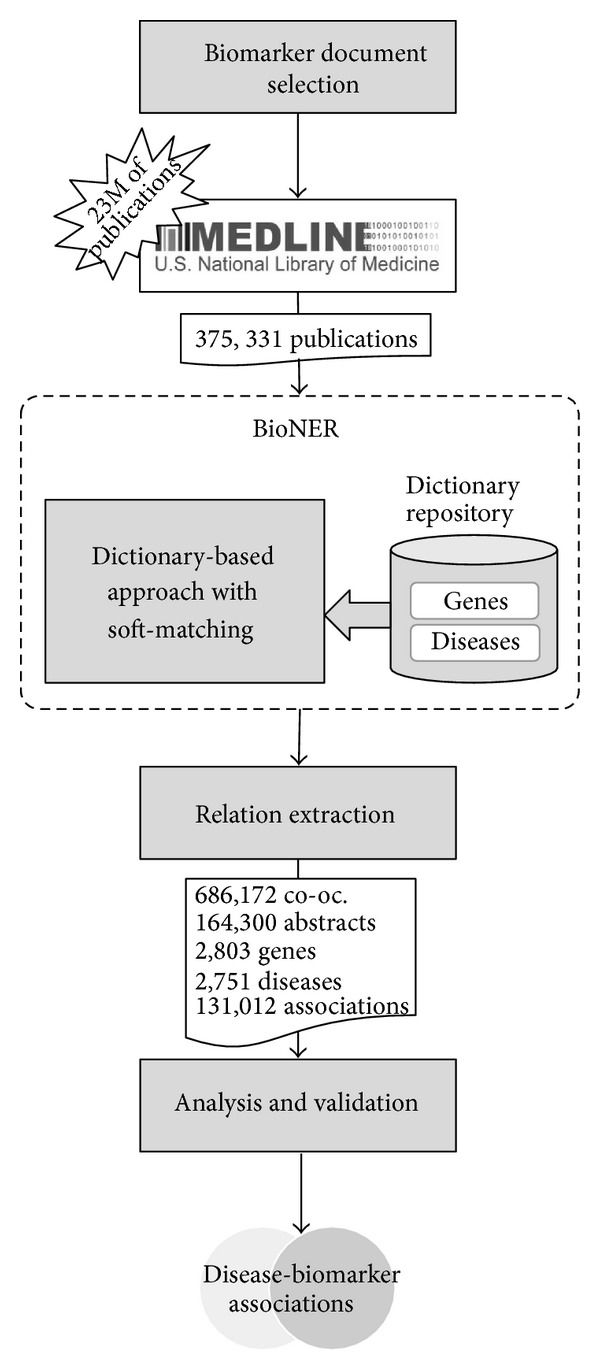
Text mining workflow.

The different steps addressed in the text mining workflow are illustrated in Figure 1 and detailed below.

### 2.1. Document Selection

To obtain a set of publications focused on biomarkers, we formulated the following PubMed query: (“Biological Markers” [MeSH Terms]) AND (has abstract [text]) AND (English [lang]) AND (“0001/01/01” [PDAT]: “2013/06/30” [PDAT]) AND “humans” [MeSH Terms], that resulted in 375,331 publications (September 30, 2013).

### 2.2. Development of Gene and Disease Dictionaries for Biomarker-Specific Information

**Figure 2:**
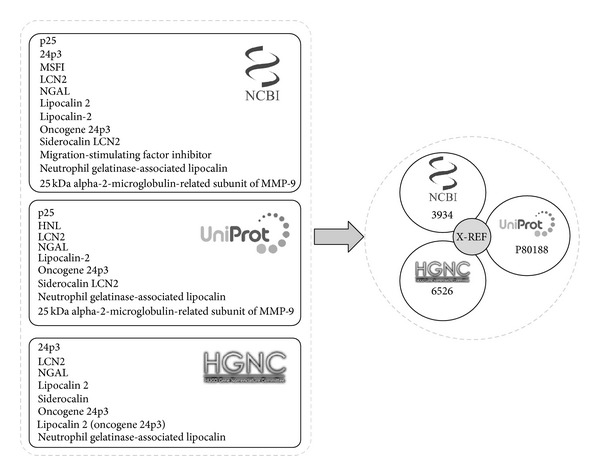
An example of the variability in terminology for genes depending on the primary sources.

*Gene Dictionary*. In order to collect the terms referring to human genes and proteins, we have integrated data from three biological databases: NCBI-Gene [17], HGNC [18], and UniProt [19, 20], followed by a semiautomatic curation process. These databases are cross-referenced between each other, providing a way to collect and integrate the terminology for a specific gene/protein entity from the different sources in a single entity.  Figure 2 shows an example of terminology integration for the Lipocalin-2 gene. Note that we do not make a distinction between gene and protein mentions in the text, because in general both types of entities share their terminology. Thus, for the sake of simplicity, we refer to genes and proteins as genes.

*Disease Dictionary*. The Unified Medical Language System (UMLS) [21] database was used to create the disease dictionary. The UMLS Metathesaurus is a large, multipurpose, and multilingual thesaurus that contains millions of biomedical and health-related concepts, their synonymous names, and their known relationships. We selected all the concepts in English from the freely distributed vocabularies corresponding to the following semantic types: Congenital Abnormality (T019), Acquired Abnormality (T020), Disease or Syndrome (T047), Mental or Behavioral Dysfunction (T048), Experimental Model of Disease (T050), Sign or Symptom (T184), Anatomical Abnormality (T190), and Neoplastic Process (T191).

Both dictionaries were curated and extended semiautomatically using different rules to facilitate the matching task. Each dictionary has its own distinctive features; for example, the gene dictionary has a high prevalence of acronyms referring to genes (i.e., A2MP1, NOTCH1, and SF3B1), whereas long terms are prevalent in the disease dictionary (i.e., Alzheimer's disease, acute lymphoblastic leukemia, primary eosinophilic endomyocardial restrictive cardiomyopathy, and rheumatic tricuspid stenosis and insufficiency). In our curation process we defined the following rules with specific adjustments depending on the dictionary.

1. To reduce ambiguity in the dictionary, the terms with a length smaller than three characters are removed.
2. A specific number of characters are replaced by their general form; that is, the characters “à, ö, ç, û” are replaced by “a, o, c, u” (i.e.,* Sjögren-Larsson syndrome* by* Sjogren-Larsson syndrome*).
3. New variants are generated for gene symbols (i.e.,* IL2*,* IL 2*,* IL *(*2),* or* IL-2* is the same acronym referring to* interleukin 2*).
4. Terms containing digits (Arabic numbers) can be written with roman numbers. New terms are generated by replacing Arabic with Roman numbers (*Adenylosuccinate lyase deficiency type 4* by* Adenylosuccinate lyase deficiency type IV*).
5. Terms can contain Greek letters (such as HP1-alpha, HP1-beta, and HP1-gamma) or symbols (as HP1-*α*, HP1-*β*, and HP1-*γ*); both cases are considered.
6. Prefix and suffix labels not used in natural language are removed from the terms (i.e.,* [X]Gastric neurosis* or* Leber Aongenital Amaurosis [Disease/Finding]* by* Gastric neurosis* and* Leber Congenital Amaurosis*).
7. All terms are transformed into lowercase characters (i.e.,* FALDH deficiency* by* faldh deficiency*).
8. All punctuation marks are removed to improve the fuzzy matching (i.e.,* hnf-3-gamma* by* hnf 3 gamma*).

Particularly, the disease dictionary was also processed with Casper [22], a rule-based software that suppresses undesired terms from the UMLS Metathesaurus and generates additional synonyms and spelling variations, and, afterwards, manually curated in order to remove very general terms such DISEASE or SYNDROME.

As a final step, to select a set of putative biomarker genes from our* gene dictionary*, we conducted a text mining search for the genes that are mentioned together with biomarker terms in the same sentence (biomarker rule filtering) and then all entries of this set of genes were extracted from the dictionary as putative biomarkers-related terms. The rationale of this approach is that genes mentioned together with terms such as “marker” are very likely biomarkers themselves. The biomarker terms were collected from the concept “biological markers” present in the MeSH terminology [11]. This step retrieved a total of 3,533 genes which were mentioned together with at least one biomarker term, and they were collected from our* gene dictionary* to create the* biomarker-specific gene dictionary*. A similar procedure was applied to obtain a subset of the* disease dictionary* relevant to the biomarkers topic, the* biomarker-specific disease dictionary*. This filter allowed the selection of 3,122 diseases cooccurring with biomarker terms.

Table 1 shows the number of concepts, the ambiguity, and the variability for all the dictionaries to illustrate the effect of the curation and rules applied. The ambiguity quantifies how a term can refer to different concepts, while the variability reflects the average number of unique terms for each concept. The best curation process is the one that improves the variability minimizing the ambiguity of the dictionaries. In the case of the* gene dictionary*, the number of terms between the raw and curated dictionaries increases in 19% with a slight effect in the ambiguity. In the case of the* disease dictionary*, there are no major changes in ambiguity and variability after dictionary curation. Overall, the curation process keeps the ambiguity and improves the variability.

### 2.3. BioNER

The BioNER system applies the biomarker-gene and disease-biomarker dictionaries using fuzzy- and pattern-matching methods to find and uniquely identify entity mentions in the literature [23–25]. Firstly, our BioNER receives the dictionary type to extract mentions and a list of document identifiers (obtained in the document selection step). Each publication is recovered from a document repository and the abstracts are split into sentences, and a set of patterns is created from the selected dictionary (*biomarker-specific gene or disease* dictionaries), after removing a list of stop words. For each sentence, the BioNER extracts the longest term from the patterns without overlap. Then, each mention is normalized to its unique identifier using the dictionary.

### 2.4. Relation Extraction

In this study, we applied a relation extraction (RE) method based on cooccurrence findings, which assumes that a biomarker and a disease are associated if they are mentioned together in the same sentence. From 164,300 abstracts, 686,172 cooccurrences were found between 2,803 biomarkers and 2,751 diseases, resulting in 131,012 disease-biomarker different associations. Certainly, the title and the body of the abstract show different writing styles, in terms of both syntax and semantics. Generally, the title or the last part of the abstract tends to express more concisely the final message of the publication, whereas the rest of the abstract contains background information and more hypothetical discourses as contextual information of the study. In order to account for these differences and make a distinction depending on where the cooccurrence is detected in the text, the system separates each abstract into 3 parts: title, abstract body, and conclusions. Then, the associations are scored based on the frequency of each association in the literature represented by a variant of the Inverse Document Frequency model [26] as follows:

(1) $$\begin{array}{c} {\text{Score}}_{\text{DB}}=idf\left(\text{DB},A\right)\cdot \overset{\left|A\right|}{\underset{i=1}{\sum }}af\left(\text{DB},A_{i}\right), \end{array}$$

(2) $$\begin{array}{c} idf\left(\text{DB},A\right)=\log_{10}\frac{\left|A\right|}{\left|\left\{a\in A\;:\text{DB}\in a\right\}\right|}, \end{array}$$

(3) $$\begin{array}{c} af\left(\text{DB},A_{i}\right)=\frac{f\left(\text{DB},A_{i}\right)}{\max\left\{f\left(XY,A_{i}\right):XY\in A_{i}\right\}}, \end{array}$$

where the score for the association between disease D and biomarker B (Score_DB_, (1)) is obtained as the product between the inverse document frequency of the association between D and B (*idf*(DB, *A*), (2)) and the normalized frequency of the association between D and B overall the documents (*af*(DB, *A*_*i*_), (3)).

The *idf* provides an indication of the popularity of the association across the corpus of documents under study, and it is obtained by dividing the total number of abstracts (|*A*|) by the number of abstracts containing the association between D and B (|{*a* ∈ *A* : DB ∈ *a*}|) and taking the logarithm of that quotient (2). The function *af*(DB, *A*_*i*_) in (3) is the frequency of the association between B and D in the *i*th abstract (*A*_*i*_) and it is defined with a quotient between *f*(DB, *A*_*i*_), which is the number of times that the association between B and D occurs in *A*_*i*_ (multiplied by 2 if DB occurs in title or conclusion of *A*_*i*_, or 1 if DB occurs in the body), and the maximum frequency of any association in *A*_*i*_ (max⁡{*f*(*XY*, *A*_*i*_) : *XY* ∈ *A*_*i*_}).

### 2.5. Analysis and Validation

In order to validate the disease-biomarker associations identified by text mining, we compared them to the biomarker information contained in the DisGeNET database, release 2.0 (July, 2012). DisGeNET is a database that integrates knowledge on the genes associated with human diseases from various expert curated databases and the literature [27, 28]. For this study we used the set of associations labelled as “biomarker” according to the DisGeNET gene-disease association ontology [29, 30]. We collected a list of 12,887 genes associated with 6,135 different disease terms stored in DisGeNET.

## 3. Results and Discussion 

In this paper, we present a new methodology to extract disease-biomarker associations from the literature. One of the major challenges that any text mining application faces is the variability of terms referring to the same concept; and then, consequently, the identification of entities in a nonambiguous manner (i.e., gene, protein, and disease). In this respect, biomedical terms gathered in domain-specific lexicons such as dictionaries, ontologies, and terms classifications (i.e., MeSH disease tree [31]) serve to organize synonymous terms into a central concept, facilitating both entity recognition and the hierarchical exploration of the results [32]. Another challenge in biomedical text mining is the identification of relationships between two entities [13]. Thus, our methodology faces both challenges by (1) the identification of* biomarker* and* disease *entities by means of the BioNER system and (2) the extraction of relationships between these entities by cooccurrence in sentences. An analysis of the associations between disease and biomarker is presented according to their mention frequency in MEDLINE, and they are evaluated by manual inspection and by comparison with the biomarker information integrated in the DisGeNET database.

The application of our text mining approach on a set of 375,331 publications pertaining to biomarkers (see Section 2.1) resulted in 686,172 disease-gene cooccurrences found in 164,300 abstracts. These cooccurrences represented associations between 2,803 genes and 2,751 diseases, giving rise to 131,012 unique disease-gene associations, which should be considered as potential disease-biomarker associations due to both the* document selection strategy* and the* biomarker rule filtering* addressed (see Section 2 for details and find examples of sentences including disease and biomarker concepts in Table 2). It is important to remark that the biomarker and disease mentions found in the text are linked to their corresponding identifiers in standard vocabularies (NCBI Gene for biomarkers and UMLS for diseases). This normalization of the entities extracted from the publications enables the unique identification of these entities and opens the possibility of integration of the extracted information with data from other standardized resources.

### 3.1. Distribution of Biomarker Information in the Biomedical Literature

**Figure 3:**
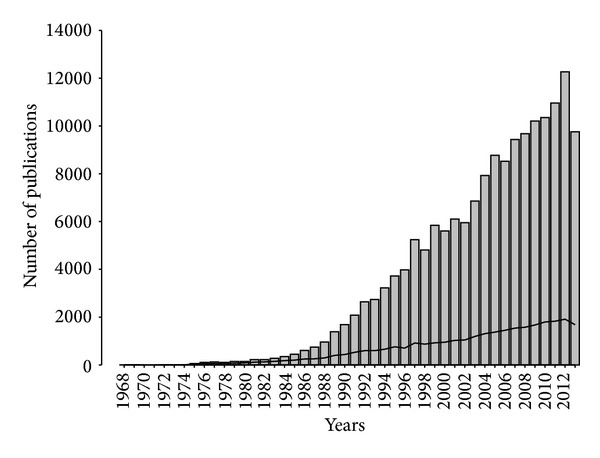
Number of publications (bars) and number of journals (line) by year.

From the approximately 23 M publications contained in the MEDLINE database, 375,331 are related to biomarkers and therefore have been annotated with the MeSH terms “biological markers” and “Biomarkers, Pharmacological” by PubMed curators. From these publications, 164,300 contain information on genes and proteins as biomarkers of a given disease in the abstract. The distribution of cooccurrences encountered in the title, the body of the abstract, and the conclusions section was 10, 85, and 5%, respectively. The evolution in reporting disease-biomarker related information throughout the years is presented in Figure 3. The document set under study represents publications in the field of biomarkers that contain information on genes and proteins from 3,983 different journals. Both the number of journals that publish disease-biomarkers-related data and the number of published articles show a progressive increase from the early 1980s. Only 5 of the journals include* marker* or* biomarker* in their journal name (*Int. J Biol. Markers* (336 abstracts),* Dis. Markers* (187),* Cancer Epidemiol. Biomarkers Prev.* (413),* Biomarkers* (11), and* Genet Test Mol. Biomarkers* (35)) and contribute to the disease-biomarker association list of this present study with a total of 2,253 disease-biomarker associations, which means only a 2% of the total list of associations identified in this present study.

**Figure 4:**
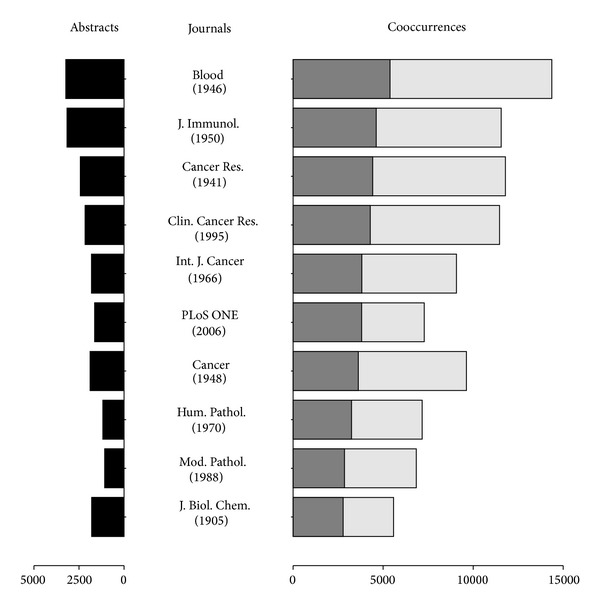
The top 10 journals sorted by unique disease-biomarker cooccurrences identified.

A further analysis of the provenance of the cooccurrences, in terms of journals that report them, was carried out and results for the top 10 journals are represented in Figure 4. Concretely, these 10 journals report 13% (94,760) of the cooccurrences identified in the 12% (20,341) of the abstracts of the working set. Interestingly, the total number of cooccurrences is proportional to the number of disease-biomarker associations recorded from each of the top 10 journals. Note that the publication start year of each journal points out that not necessarily those journals reporting most disease-biomarker associations in our working set started their publication earlier than others (i.e., Plos ONE). Most of the articles published in these top 10 journals describe basic laboratory, translational, and clinical investigations, and some of them have a special focus on specific therapeutic areas: hematology (*Blood*), immunology (*J Immunol.*), and oncology (*Cancer Res., Clin. Cancer Res., Cancer*,* Int. J Cancer*). In fact, over 300 journals of the list include the “clinical” word in their name, over 200 include the word “cancer” or the prefix “onco”, and around 140 include the prefix “immun”; which are by far the main fields where biomarkers are being investigated.

Twenty-one percent of the disease-biomarker associations were identified in the top 10 journals (56% of diseases and 68% of biomarkers collected in this study, resp.; see Figure 4). Over 50% of the associations are retrieved from the first 100 journals (81% of diseases and 87% of biomarkers), and over 80% are from the first 500 journals (95% of diseases and 97% of biomarkers); and till we consider the first 1,000 journals we do not reach more than 90% of the total associations (98% of diseases and 99% of biomarkers).

This analysis shows that the number of journals and articles that report biomarkers information has increased during the last years, and this fact (i) expands the publication bias (few journals are specialized in biomarkers research and development, while most of journals include in their scope the biomarker topic or at least publish special issues devoted to biomarkers research), (ii) makes difficult the retrieval and exploitation of this information, and (iii) highlights the need of an improvement in the biomarker related data reporting [33] to ensure better quality of automatic extraction by means of mining techniques.

### 3.2. Analysis and Validation of the Disease-Biomarker Associations

The 131,012 disease-biomarker associations were scored based on their mention frequency in MEDLINE (see Table 3 for details of the associations distribution based on the score described in Section 2.4). The top 10 associations with higher score are shown in Table 3, where very well-studied disease biomarkers can be found (for instance, TP53 and ERBB2 for cancer and CD4 for immunodeficiencies).

**Figure 5:**
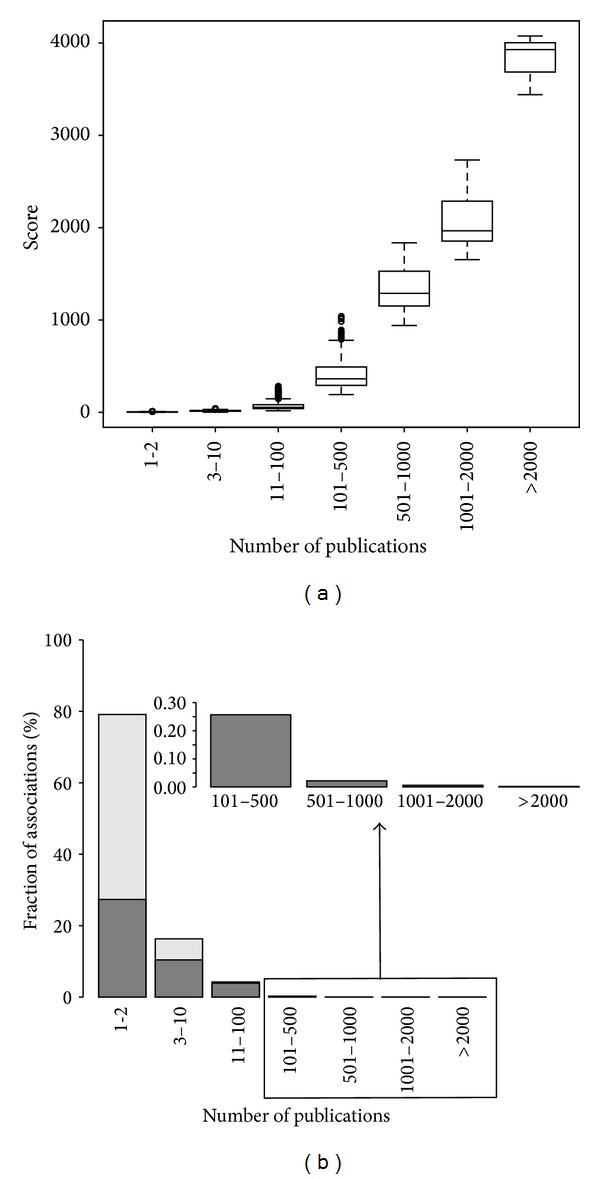
Associations analysis. (a) Boxplot showing the score* versus* number of publications supporting each disease-biomarker association. (b) Distribution of associations based on the number of publications that support each association. The fraction of the associations that were reported in the last three years is highlighted as dark grey bars.

Figure 5 shows the analysis of the associations based on the Score_DB_ (a) and the number of publications (b). The percentage of associations reported by different numbers of publications (from 1-2 publications to more than 2,000) in the corpus under study (131,012 associations, light grey bars) is represented. The caption shows the data for the associations reported by more than 100 publications, which represent a small percentage of all the associations. Note that most of these associations (more than 90%) have been reported in publications from the last three years.

In general, associations with high score are supported by a high number of publications (Figure 5(a)), and globally around 80% of the associations are supported by only 1 or 2 publications and have a low score. From this set, 35% corresponds to studies published in the last 3 years (Figure 5(b)). The novelty of these associations could explain the low number of supporting articles. Thus, it is likely that the remaining 65% of the associations supported by very few publications represent studies that could no longer be reproduced or are focused on very specific genes or diseases that are not of widespread interest, as in the case of the most prevalent diseases such as some types of neoplasms. It is noteworthy that, for most of the associations supported by more than 10 articles, at least one of these articles has been published in the last 3 years (Figure 5(b)).

**Figure 6:**
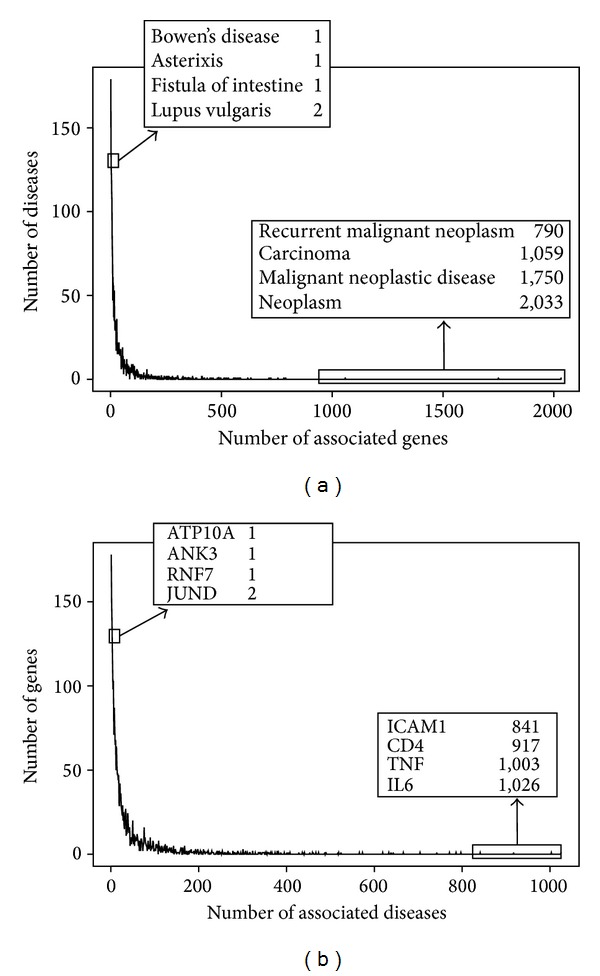
Distribution of the number of associated biomarkers (for diseases, (a)) and diseases (for biomarkers, (b)). Gene symbols from HGNC are used for the biomarkers.

A further analysis of the results allows us to identify both the set of biomarkers associated with a large number of diseases (see Table 4) and the set of biomarkers associated with few (1 or 2) diseases. This information can be an indication of the “specificity” of a biomarker with respect to diseases. For example, a biomarker associated with many different diseases would be less specific than the other that has been studied in relation to a single disease, and,* vice versa*, the same consideration can be done for the diseases. The distribution of the number of associated diseases (for biomarkers) and biomarkers (for diseases) is depicted in Figure 6. For example, the genes PANK2, ANK3, and RNF7 appear as very specific biomarkers as they are associated with a single disease. On the other hand, several genes related to immune responses have been reported in associations with hundreds of diseases, such as IL6, TNF, and CD4 (Table 4 and Figure 6).

With respect to diseases, the results show that cancer is the therapeutic area that has more associated biomarkers (Table 5 and Figure 6). For instance, leukemia is associated with 782 biomarkers, and some of them (NOTCH1, SF3B1, and BIRC3) have been found in recent literature reporting [34]. In contrast, few biomarkers have been identified for diseases like lupus vulgaris and Bowen's disease.

The disease-biomarker associations were also assessed according to the disease classes of the MeSH disease classification [31], indicating that neoplasm, nervous system diseases, and immune system diseases are by far the ones more investigated in the biomarkers research field (see Table 6).

In average, 11% of the disease-biomarker associations identified by our text mining approach were found in DisGeNET. Since DisGeNET contains information on the genetic determinants of human diseases and is not specially focused on biomarkers as defined in the present study, it is not surprising that only a small fraction of the information extracted from the literature is contained in DisGeNET database (July 2012 release). In addition, lag time in the population of the source databases by human curators may account for this difference. The dataset provided by the text mining approach here presented constitutes a large and valuable source of information on disease-related biomarkers, which can be used to populate specialized databases and to guide further research on biomarker validation. However, it is important to note that, based on the relation extraction approach used in this study, a proportion of the disease-biomarker associations found by this approach could be false positives. Future work will take in consideration the syntactic structure of the sentences in which a biomarker and a disease cooccur for the relation extraction process, with the aim of improving the precision of the approach. Search of semantic patterns reported in the abstracts' sentences will be checked in parallel to new data available from current and new disease-related biomarkers databases, with the aim of providing comprehensive and up-to-date knowledge to those biomedical researchers working in the disease-related biomarker field.

### 3.3. Related Work

Only few studies have proposed text mining approaches for extraction of biomarker related data [14–16]. For example, Younesi et al. presented a methodology for the retrieval of documents about biomarkers and showed as use cases the identification of markers for Alzheimer disease and multiple sclerosis [14]. Hui and Chunmei propose a finite state machine to identify pathways and diseases related to biomarkers [15]. We show in this study that a knowledge-driven approach is able to systematically exploit biomarker-specific information from large literature databases (e.g., MEDLINE) providing a comprehensive resource of biomarkers associated with diseases covering all the therapeutic areas.

## 4. Conclusions and Future Directions

The biomedical literature represents a rich resource for the identification of biomarker related information. However, both the size of the literature databases and the lack of standardization make difficult the automatic exploitation of the information contained in these resources. Text mining approaches have proven to be useful for the extraction of relations between entities, especially for the identification of interactions between proteins [13]. Here, we show that a knowledge-driven text mining approach can exploit a large literature database to extract a dataset of biomarkers related to diseases covering all therapeutic areas.

A bibliometric analysis of the journals reporting biomarker related information during the last 40 years highlighted the disparity among journals of different disciplines which expands the publication bias, hampers the information retrieval and its exploitation, and, even, evidences the need of a standardization of the biomarker related data reporting to improve the quality of automatic extraction by means of mining techniques and gain confidence with their outcomes.

Our methodology focused on the extraction of disease-biomarker associations reported in the literature. This knowledge-driven approach takes advantage of the annotation of MEDLINE publications pertaining to biomarkers with MeSH terms, narrowing the search for specific publications and therefore minimizing the false positive ratio. The application of this methodology resulted in the identification of 131,012 disease-biomarker associations between 2,803 genes and 2,751 diseases and represents a valuable knowledge base for those interested in disease-related biomarkers. The results of this present study are available at http://ibi.imim.es/biomarkers/.

Future work in this area will focus on the identification of the type of association between the disease and the biomarker (for instance, distinguishing between the different levels of certainty that can be used to express an association or to specify the type of molecular change of the gene or protein associated with the disease). In addition, other types of molecules that can act as disease biomarkers could be identified as well.

## Figures and Tables

**Table 1:** Contents and statistics of gene, disease, and biomarker-gene and disease-biomarker dictionaries.

Dictionary	Number of concepts	Number of terms	Ambiguity	Variability
Gene	50,090	545,519	1.51	5.98
Gene curated/extended	50,090	649,414	1.46	12.96
Disease	79,781	378,616	1.01	4.14
Disease curated/extended	74,073	294,371	1.02	3.97
Biomarker-specific gene	3,533	89,236	1.27	25.26
Biomarker-specific disease	3,122	35,686	1.05	11.43

**Table 2:** Examples of sentences including disease-biomarker cooccurrences.

Disease (CUI)^a^	Biomarker (Gene ID)^b^	PMID (year)	Sentence
Hodgkin's lymphoma (C0019829)	Anti-Mullerian hormone (268)	17726078 (2007)	**Anti-Mullerian hormone** is a sensitive *serum marker* for gonadal function in women treated for **Hodgkin's lymphoma** during childhood. (TITLE)
TCC (C1861305)	CK20 (54474)	17397492 (2007)	**CK20** is an important *biomarker* that can be used to identify **TCC** in urine cytology smears. (CONCLUSIONS)
Autism (C0004352)	Brain-derived neurotrophic factor (BDNF) (627)	19119429 (2008)	To investigate levels of **brain-derived neurotrophic factor **(**BDNF**) in midpregnancy and neonatal blood specimens as early *biologic markers* for **autism**; we conducted a population-based case-control study nested within the cohort of infants born from July 2000 to September 2001 to women who participated in the prenatal screening program in Orange County, CA. (BODY)
Acute kidney injury (AKI) (C0022660)	Neutrophil gelatinase-associated lipocalin (NGAL) (3934), netrin-1 (9423)	21740336 (2011)	**Neutrophil gelatinase-associated lipocalin** (**NGAL**) and **netrin-1** have been proposed over the past years as emergent *biomarkers* for the early and accurate diagnosis and monitoring of **acute kidney injury** (**AKI**). (BODY)
Chronic heart failure (C0264716)	Cardiac troponin I (7137)	21751783 (2011)	Top-down quantitative proteomics identified phosphorylation of **cardiac troponin I** as a candidate *biomarker* for **chronic heart failure**. (TITLE)
Adenocarcinomas (C0001418)	MOC-31 (4072)	21732548 (2011)	**MOC-31** is an established *immunologic marker* to detect **adenocarcinomas**. (BODY)
Lung adenocarcinoma (C0152013)	ROM (6094)	21748260 (2012)	Hence, serum **ROM** level may be a useful *biomarker* for staging of **lung adenocarcinoma**. (BODY)

^a^Concept unique identifier at UMLS.

^b^NCBI gene identifier.

**Table 3:** The top 10 disease-biomarker associations. Disease-biomarker associations were ranked according to Score_DB_ (see Section 2 for more details). The complete list of the associations is available at http://ibi.imim.es/biomarkers/.

Score	Disease name (CUI^a^)	Gene symbol (Gene ID^b^)	Number of abstracts
4076.42	NEOPLASM (C0027651)	TP53 (7157)	3,042
3930.25	NEOPLASM (C0027651)	ERBB2 (2064)	2,582
3441.32	NEOPLASM (C0027651)	CEACAM5 (1048)	2,234
2733.92	IMMUNODEFICIENCY DISORDER (C0021051)	CD4 (920)	1,548
2546.27	NEOPLASM (C0027651)	EGFR (1956)	1,710
2028.21	LEUKEMIA (C0023418)	CD34 (947)	1,071
1988.57	NEOPLASM (C0027651)	ESR1 (2099)	1,179
1943.15	NEOPLASM (C0027651)	AFP (174)	1,169
1915.15	NEOPLASM (C0027651)	CD34 (947)	1,108
1836.03	MALIGNANT NEOPLASTIC DISEASE (C0006826)	KLK3 (354)	936

^a^Concept unique identifier at UMLS.

^b^NCBI gene identifier.

**Table 4:** The top 10 genes sorted by the number of associated diseases. The complete list of genes is available at http://ibi.imim.es/biomarkers/.

Gene symbol	Gene ID^a^	Gene name	Number of diseases
IL6	3569	Interleukin 6 (interferon, beta 2)	1,025
TNF	7124	Tumor necrosis factor	1,003
CD4	920	CD4 molecule	917
ICAM1	3383	Intercellular adhesion molecule 1	841
TP53	7157	Tumor protein p53	797
CRP	1401	C-reactive protein, pentraxin-related	786
CD8A	925	CD8a molecule	771
CD34	947	CD34 molecule	742
VEGFA	7422	Vascular endothelial growth factor A	704
ACE	1636	Angiotensin I converting enzyme	666

^a^NCBI gene identifier.

**Table 5:** The top 10 diseases sorted by the number of associated biomarkers. The complete list of diseases is available at http://ibi.imim.es/biomarkers/.

Disease name	CUI^a^	Number of genes
Neoplasm	C0027651	2,033
Malignant neoplastic disease	C0006826	1,750
Carcinoma	C0007097	1,059
Recurrent malignant neoplasm	C1458156	790
Leukemia	C0023418	782
Malignant melanoma	C0025202	755
Liver cell carcinoma	C2239176	723
Congenital deformity	C0000768	715
Tumor angiogenesis	C1519670	633
Tumor progression	C1519176	619

^a^Concept unique identifier at UMLS.

**Table 6:** Comparison of disease-biomarkers pairs identified by the text mining (TM) approach with disease-biomarkers annotations in DisGeNET, based on MeSH disease classification [31].

MeSH disease class	MeSH disease class name	Number of disease-biomarker associations	The number validated with DisGeNET (%)
C01	Bacterial infections and mycoses	1,529	164 (10.73)
C02	Virus diseases	3,297	302 (9.16)
C03	Parasitic diseases	590	82 (13.90)
**C04**	**Neoplasms**	**31,627**	**5,264 (16.64)**
C05	Musculoskeletal diseases	5,771	388 (6.72)
C06	Digestive system diseases	8,154	1,156 (14.18)
C07	Stomatognathic diseases	2,531	195 (7.70)
C08	Respiratory tract diseases	5,460	735 (13.46)
C09	Otorhinolaryngologic diseases	770	40 (5.19)
**C10**	**Nervous system diseases**	**10,819**	**1,132 (10.46)**
C11	Eye diseases	2,513	226 (8.99)
C12	Male urogenital diseases	5,110	666 (13.03)
C13	Female urogenital diseases and pregnancy complications	6,432	863 (13.42)
**C14**	**Cardiovascular diseases**	**9,310**	**1,393 (14.96)**
C15	Hemic and lymphatic diseases	7,689	948 (12.33)
**C16**	**Congenital, hereditary, and neonatal diseases and abnormalities**	**10,382**	**397 (3.82)**
C17	Skin and connective tissue diseases	6,724	851 (12.66)
C18	Nutritional and metabolic diseases	6,314	711 (11.26)
C19	Endocrine system diseases	5,253	681 (12.96)
**C20**	**Immune system diseases **	**10,210**	**1,393 (13.64)**
C21	Disorders of environmental origin	2	0 (0.00)
C23	Pathological conditions, signs, and symptoms	8,212	606 (7.38)
C24	Occupational diseases	72	11 (15.28)
F01	Behavior and behavior mechanisms	594	24 (4.04)
F03	Mental disorders	2,810	613 (21.89)
